# Additive Manufacturing in Dentistry: A Comparative Study of Polymeric Surgical Guide Fabrication

**DOI:** 10.3390/polym17202764

**Published:** 2025-10-15

**Authors:** Joshua García Montagut, Ana González, Rubén Paz, Luis Suárez, Pablo Bordón, Zaida Ortega, Iulian Antoniac, Ilaria Cacciotti, Adriana Ileana, Mario Monzón

**Affiliations:** 1 Mechanical Engineering Department, Edificio de Ingenierías, Campus de Tafira Baja, University of Las Palmas de Gran Canaria, 35017 Las Palmas, Spain; joshua.garcia@ulpgc.es (J.G.M.); ana.rodriguez@ulpgc.es (A.G.); ruben.paz@ulpgc.es (R.P.); luis.suarez@ulpgc.es (L.S.); pablo.bordon@ulpgc.es (P.B.); 2 Process Engineering Department, Edificio de Ingenierías, Campus de Tafira Baja, University of Las Palmas de Gran Canaria, 35017 Las Palmas, Spain; zaida.ortega@ulpgc.es; 3 Department of Metallic Materials Science, Faculty of Materials Science and Engineering, National University of Science and Technology Politehnica Bucharest, 060042 Bucharest, Romania; antoniac.iulian@gmail.com (I.A.); ileanadriana@gmail.com (A.I.); 4 INSTM Research Unit, Department of Engineering, University Niccolò Cusano, via Don Carlo Gnocchi 3, 00166 Rome, Italy; ilaria.cacciotti@unicusano.it

**Keywords:** additive manufacturing, surgical guide, dentistry, dimensional analysis

## Abstract

Additive manufacturing (AM), or 3D printing, has revolutionized surgical guide fabrication in dentistry by enabling the creation of complex, customized parts. This study aims to evaluate and compare three predominant AM technologies for polymers—Material Extrusion (MEX), Vat Photopolymerization (VPP), and Powder Bed Fusion (PBF)—for producing surgical guides, focusing on desktop-level equipment. The analysis centers on key criteria: dimensional accuracy, manufacturing time, process complexity, and cost, both for single-set and multiple-set productions. The results reveal that while VPP and MEX technologies offer sufficient dimensional accuracy for clinical use, PBF technology falls short in this regard. In terms of cost and time, VPP proves to be the most efficient technology for manufacturing multiple sets of guides, a common scenario in dental clinics. However, MEX technology demonstrates its competitiveness, particularly in single-set, on-demand fabrication due to its fast-processing time and the potential for lower material costs with proper material selection. The study concludes that while VPP has been the traditional choice, advancements have made MEX a viable and practical option for a rapid and easy integration into smaller dental clinics.

## 1. Introduction

Additive manufacturing (AM), commonly known as 3D printing, has evolved significantly since its inception with the first related patent granted to Charles W. Hull in 1986 [1]. It is capable of manufacturing a real object from a digital document by joining materials layer by layer, which differentiates it from traditional subtractive technologies. Additive manufacturing presents a multitude of advantages in medicine [2]; focusing on dental field, the principal benefits are the manufacturing of geometrical complex parts without affecting costs (as is the case with substrative technology) [3], product customisation [4], internal characteristics, and functions integration [5], and the easy use of biocompatible materials [6].

Additive manufacturing is able to work with three types of materials: metals and alloys, polymers and ceramics. The most commonly used materials are photopolymer resins, polyether ether ketone (PEEK), polylactic acid (PLA), Acrylonitrile-butadiene-styrene (ABS), metals such as titanium or stainless steel, and ceramics such as zirconium oxide, alumina, or hydroxyapatite. In addition to the above options, there are also material alloys, which are widely used in additive manufacturing [7,8,9,10,11,12,13,14].

**Figure 1:**
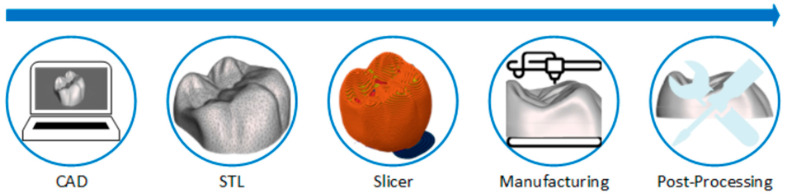
Additive manufacturing workflow.

All AM technologies follow the same workflow, as illustrated in Figure 1. The initial stage involves the digital 3D design of the component using CAD modeling software. Regardless of the specific software employed for modeling, this information is subsequently exported to a surface file, typically in STL format. In this format, the part is represented by an array of triangular facets that collectively define its entire surface. It is crucial to remember that AM operates on a layer-by-layer principle. Therefore, this 3D part must undergo a slicing process. For this, a group of specialized software known as “Slicers” is utilized. Within these slicers, printing parameters are configured, and the various layers that will ultimately constitute the complete part are generated. Following this, the part proceeds to be manufactured. It is in the manufacturing process where the process changes depending on the chosen additive manufacturing technology. Upon completion of the manufacturing process, it undergoes post-processing if necessary, yielding the final component.

The ISO/ASTM 52900:2021 standard [15] establishes a total of seven types of technologies. However, in dentistry, the predominant ones for plastics are [16] Vat Photopolymerization (VPP), Material Extrusion (MEX), and Powder Bed Fusion (PBF).

**Figure 2:**
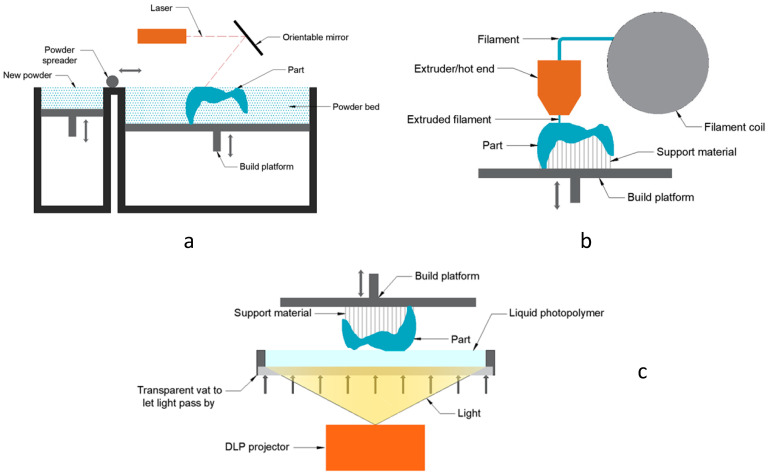
Additive manufacturing technologies: Powder Bed Fusion schema (**a**), Material Extrusion schema (**b**), and Vat Photopolymerization schema (**c**).

Vat Photopolymerization (Figure 2c) uses a liquid photopolymer resin cured by a light beam. Depending on the light source, we differentiate between Stereolithography (SLA) [17] and Digital Light Processing technology (DLP). This technology offers good resolution, good finishes, a variety of materials, and a relatively good manufacturing speed [18,19,20,21]. The materials used are usually polymers, composites (carbon fiber, Kevlar), and ceramics (alumina, zirconia, silicon carbide). However, its build volume is usually limited, and its operation is somewhat complex in terms of handling resins, although some brands try to simplify the process as much as possible.

Material Extrusion (Figure 2b) is one of the most widely accepted technologies in the general market [22]. This technology melts a thermoplastic material, which is extruded through a nozzle to create a three-dimensional object. While it is an easily accessible technology, the print volumes can be quite large, and within the range of materials to be used, there are different options for resistance and characteristics. Despite being less automated due to the laminate configuration, which has many variables, its resolution and finish are sufficient for many uses [23].

Powder Bed Fusion (Figure 2a) is a technology based on the sintering or melting of a powder bed, which grows layer by layer, finally forming the 3D part (Figure 2). As with VPP technology, depending on how we sinter or melt the powder, three main technologies can be identified: Selective Laser Sintering (SLS) for polymers or metals, Selective Laser Melting (SLM) for metals, or Electron Beam Melting (EBM) for metals. Among its advantages are the lack of need for supports (especially in SLS), allowing for better use of print volumes, the easy reuse of unsintered material, its dimensional accuracy, and its wide range of materials. On the other hand, this technology is still relatively slow and necessarily requires post-processing, thus complicating its operation.

Additive manufacturing technology, in general, has gained relevance in the modern dental sector due to its ability to manufacture complex, unique, and customized parts for each patient [24]. Some examples of them are dental models, surgical guides, splints, provisional restorations, and dental implants [25]. In Table 1, we can see examples of use in dentistry divided by AM technologies for polymers.

One of the personalized parts manufactured in this field is the surgical guides. These parts are designed after using medical images of the patient and planning the surgery. Its main objective is to fit the patient’s mandibula to serve as a guide so that the different surgical procedures are well-positioned in all planes and axes with respect to the patient.

Traditionally, these pieces are manufactured by subtractive manufacturing (principally Computerized Numerical Control—CNC), but authors such as Henprasert et al. [40] demonstrated that there are no significant differences between the additive and subtractive manufacturing processes from a dimensional accuracy point of view, specifically for surgical guides production. Jeon et al. [41] went a step further and affirmed that additive manufacturing is even better in some cases, being quick with high quality, and at a very good cost-effectiveness ratio [28,29,42].

Authors such as Rouzé l’Alzit et al. [43] and others [17,19,35] focused their research on the precision and resilience of different additive manufacturing technologies for the production of dental sector parts, with their differences increasingly narrowing as they improve. Authors such as Sun et al. [32] demonstrated that there are no significant dimensional differences directly comparing the MEX and VPP technologies for manufacturing surgical guides.

Moreover, another factor must be taken into account when opting for one technology or another. Globally, 69% of dental services are offered by dental clinics [44]. Taking into account the size of the market [45] and the number of small clinics, it is estimated that most dental clinics do not reach EUR 500,000 in annual turnover. In Spain, at least half of dental clinics consist of three offices or fewer [46]; among these, more than 40% have a turnover of less than EUR 250,000 a year, and only 18% reach a million euros in turnover [46]. This means that, on many occasions, the cost of acquisition and its learning curve are key factors when choosing a manufacturing technology, not so much its suitability. In terms of cost-efficiency, Milijanovic et al. [37] established the following order from best to worst: SLA, material jetting, MEX, SLS, SLM, and EBM.

In this study, it is proposed to manufacture a case study through the three additive manufacturing technologies for polymers mentioned above (MEX, SLA, and SLS) using exclusively desktop equipment in the low-cost range. The main aim is to compare their dimensional accuracy, manufacturing costs, including different materials, process complexity, and time required.

## 2. Materials and Methods

### 2.1. Selected Surgical Guide Model

**Figure 3:**
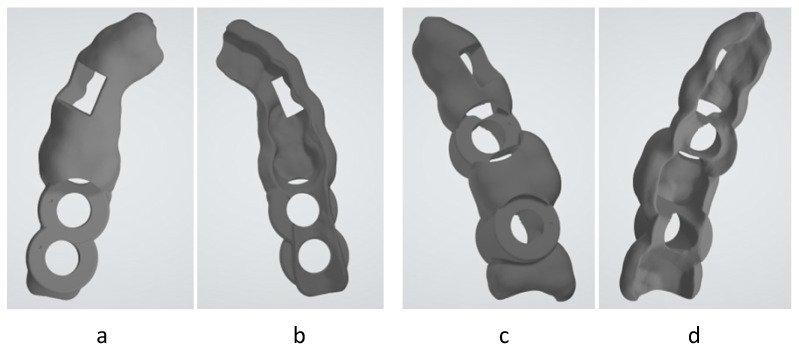
Surgical guide 1 front (**a**) and rear (**b**) view, and surgical guide 2 front (**c**) and rear (**d**) view.

For the comparison of the different technologies, a surgical guide commonly used in dentistry has been chosen as the study object. Each guide has a different correct positioning and different needs of supports and adhesion configuration; because of that, two surgical guide designs (Figure 3) were used in this study. In Figure 3a,c, the top views of each of the designs can be seen, while Figure 3b,d show their bottom views.

The temporal fixation of the guide over the teeth guarantees the correct position and angle of subsequent implants using the health teeth as reference. In guide number 1, two holes are designed with a diameter of 6 mm and are between a separation by 8.05 mm. For the guide number 2, there are two 6 mm holes with a distance between them of 12.28 mm. In contrast to the first guide, where the holes were in a row, the second guide has a healthy tooth in between.

In each printing step, one unit of every guide is manufactured, with these being manufactured at the same time. Guide number 1 occupies a manufacturing space of 22.93 × 47.5 × 13.43 mm, and a material volume of 1791.28 mm^3^. In the case of the second guide, we have a manufacturing space of 28.22 × 48.52 × 16.18 mm, and a material volume of 2980.43 mm^3^.

### 2.2. Additive Manufacturing Equipment

The equipment selected in this study was chosen for easy acquisition, installation, and use. Three different additive manufacturing machines were selected.

For the MEX technology, a Creatbot Peek-300 (Creatbot, Zhengzhou City, Henan Province, China) was used. It is a desktop machine with a print volume of 300 × 300 × 400 mm^3^ and the ability to work with technical materials such as ABS, PP, or PEEK with a high melting temperature. It is able to automatically set the extruder, bed, and chamber temperatures, but it needs to manually adjust the hot-air flow and the filament preheat temperatures. This machine uses its own laminator with setups pre-configured for different materials; however, normally, a manual calibration process is needed for each material.

In the case of PBF technology, a Sinterit Lisa Pro was chosen (Sinterit, Kraków, Poland). This is not a desktop machine, but presents reduced total dimensions (690 × 500 × 880 mm), making it suitable for small laboratories. With print dimensions of 110 × 160 × 230 mm, it is mainly focused on working with polyamides. In this case, even with the possibility to change 32 parameters, the pre-configured setups make the lamination process semiautomated. However, the manufacturing process gets complicated due to the step of preparing the powder before manufacture and extracting the parts after (cleaning and sandblasting).

For the SLA technology, the chosen machine was Formlabs Form 2 (Formlabs, Somerville, MA, USA). This is a desktop machine with a print dimension of 145 × 145 × 175 mm. This machine has a multitude of materials to work with, including some specific to the dental sector. In addition, its slicing software is almost automatic, with a specific variant for dental applications. For this case, the core of the printed object is not completely solid at the end of the manufacturing process, so a post-process of washing and curing with a specific machine (Form Cure) is needed.

### 2.3. Selected Materials

Although each of the selected technologies is capable of working with different polymeric materials, this study focused on one material commonly used in dentistry for each technology.

For MEX technology, and exploiting the special temperature capability of the CreatBot, the Polyetheretherketone, PEEK K10 Enterprise from the brand Kexcelled (Suzhou, China), in 1.75 mm diameter wire format and natural color was selected. PEEK is a high-performance polymer known for its exceptional strength, biocompatibility, and thermal stability. In dentistry, it is used to create implants, prosthetics, and custom devices that mimic bone’s mechanical properties and reduce metal allergies [47].

For PBF technology, i.e., Sinterit ecosystem, the polyamide Sinterit PA12 smooth material was chosen. It is provided in powder format with a diameter between 19 and 90 µm and a grey color. PA12 is a thermoplastic material known for its high strength, durability, flexibility, and biocompatibility. In dentistry, it is used to create accurate dental models, surgical guides, dental aligners, and prosthetic and orthodontic components, offering lightweight parts with abrasion and chemical resistance [48].

For the VPP technology, and because the chosen machine has a closed material catalogue, the photopolymeric Formlabs White Resin V4 was chosen. It is a highly valued material for its matte finish, complete opacity, and excellent ability to reproduce tiny details. In dentistry, this makes it a perfect choice for producing study models, molds for creating clear aligners, and anatomical or fit-testing prototypes where visual and surface accuracy are crucial [49]. Table 2 shows a comparison between the mechanical properties of the selected materials under the most favorable direction of build (data from the supplier material datasheet).

### 2.4. Configuration of Manufacturing Processes

One of the common processes, but not the same for all technologies, is slicing. Each machine uses its own software and its own configuration parameters. Because the geometrical configuration parameters of each machine can vary the results of the comparison, it has been decided that the same geometrical manufacturing parameters were set up for all the selected machines: a part density of 100% and a layer height of 0.1 mm.

The CreatWare V7.2.1 lamination software is used for the Creatbot machine (MEX technology). For the manufacturing with PEEK material, the following set temperatures were set: extruder (420 °C), bed (120 °C), chamber (70 °C), hot-air (120 °C), and filament chamber heating (70 °C). Also, the use of skirts and supports was activated.

The Sinterit machine uses Sinterit Studio V1.10.9 as lamination software. As mentioned above, the configuration is already predefined for each manufacturer’s material (melting point of 185 °C and heat deflection temperature of 68 °C for Sinterit PA12 smooth material), and supports are not needed.

In the case of the VPP technology, the software used is Preform V3.52.1. In this case, the use of supports and a raft was also necessary, but the rest of the parameters were automatically configured, choosing the material: a chamber temperature of 33 °C for Formlabs White Resin V4. For post-curing, a first-generation Formlabs Cure machine was used for 30 min at 60 °C.

### 2.5. Accuracy and Dimensional Study

Depending on the technology and parameter configuration used, there can be small dimensional differences between the original design and the manufactured part. While the suggests that all three technologies are good options in dentistry, with the use of low-cost machines and equipment, this affirmation is not so clear. Thus, in this study, the dimensional accuracy was taken into account, comparing the printed surgical guide number 1.

**Figure 4:**
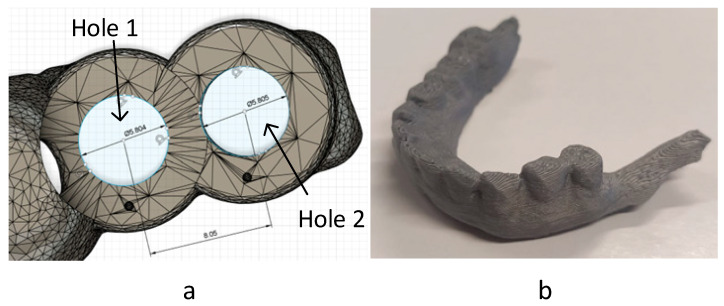
Surgical guide 1: reference measurements (**a**) and jaw from case reproduction (**b**).

The first test was a measurement check. The hole diameters and the distance between them were measured (one per dimension), using a profile projector (Nikon V-12A, Tokyo, Japan) with an accuracy of 3.0 + L/50 µm, and compared with the original design (Figure 4a).

To complement the previous test, a pass/fail test was carried out to confirm the results of the first test. This is done to ensure that, even with varying degrees of precision, the machines are capable of manufacturing parts that meet our dimensional requirements. The real jaw from the case was reproduced, and checked whether the guide, manufactured in each technology, fitted correctly with the jaw (Figure 4b). This jaw was manufactured by MEX in ABS material, as it was the simplest and most economical technology/material available to have a replica with an acceptable accuracy.

### 2.6. Methodology for Cost Analysis

Once the parts were manufactured, the comparative study was carried out by measuring two output parameters: time and production cost.

To measure the manufacturing times, the work times of the operator were considered, as well as the work times of the machine. In turn, each of them was divided into preparation time, manufacturing time, extraction time, and post-processing time. Each technology has its prior preparations and post-manufacturing work, which are also taken into account:

- MEX: space cleaning, slicing, material feeding, machine start-up, and support removal.
- PBF: space cleaning, slicing, material feeding, machine start-up, part extraction from the powder block, and sandblasting cleaning.
- VPP: space cleaning, slicing, material feeding, machine start-up, washing in isopropyl alcohol, post-wash curing, and support removal.

To measure the costs, fixed and variable factors were considered, taking two parameters as a reference for comparison: machine use cost and operator time cost, both per minute.

- Machine use cost: The acquisition price of each technology (*CA*) was established, and this was used as a reference to define the maintenance and other costs during its useful life (*CM*). We estimated this cost at 10% of the total cost of the machine.

To calculate the depreciation of the machine, it was considered that they work 24 h, 247 days a year, and must be depreciated in 5 years. Therefore, to calculate the machine use cost per minute of use (*CU)*, we used Equation (1).

(1) $$CU\;(€/min)=\frac{CA\;\left(€\right)+CM(€)}{5\;years\;x\;247\;days\;x\;24\;hours\;x\;60\;minutes}$$

In addition, another important cost to take into account in the use of the machinery is the electrical cost (*CE*), which was set at 0.25 EUR/kWh. The total cost varies in each machine depending on its operating electrical consumption (*CEh*), calculating the electrical cost per minute (*CEm*) according to Equation (2). To consider the most unfavorable scenario, the maximum power consumption of the machine at any given time was used to measure power consumption.

(2) $$CEm\;(€/min)=\frac{CE\;\left(€\right)x\;CEh\;(€)}{60}$$

- As for the operator cost (CO), the salary tables of the collective bargaining agreement will be used as a reference, establishing the company cost of the operator at 12.04 EUR/h (0.2 EUR/min).

For this analysis, it was considered that the costs calculated before do not significantly depend on the material, but on the technology; so, the cost of the material was introduced as an additional variable in the comparative analysis. To study how the material costs affect the comparison, the final cost for different materials was analyzed, adding this cost to all the others previously considered. To calculate this material cost (CMat), we used Equation (3), where *Vm* is the volume of material used in cm^3^ and *PVm* is the sale price in euros per cm^3^ of material.

(3) CMat (€/min)=Vm x PVm

Finally, the final manufacturing cost (CF) was given by Equation (4).

(4) $$CF=\sum {\left(CU+CEm\right)}_{i}x\;{Tm}_{i}+CO\;x\;To+CMat$$

where *Tm* and *To* are the machine and operator use times in minutes, respectively. The subscript i indicates the different machines used in the process of each technology.

Up to this point, all these comparisons have been made for the manufacturing of a single set of parts; however, taking into account the maximum working capacity of each machine, we can make more than one set of parts per print. In this way, the preparation and extraction times would remain the same, but the manufacturing and post-production times would be increased in proportion to the number of parts.

The increase in manufacturing time was indicated directly in the slicer of each technology. Post-production times were increased by 75% of the time used for a single unit, multiplied by as many extra parts as we can manufacture; all this according to Equation (5).

(5) $$T_{t}=T_{i}x\;(1+0^{\prime }75x(N-1))$$

where $T_{t}$ and $T_{i}$ are the total time and the time used when a single set is printed, respectively (in minutes), and N is the total number of sets manufactured (one set is a copy of each surgical guide).

With this information, we calculated the manufacturing cost per individual set, which is another comparison factor.

### 2.7. Manufacturing Processability

To measure the complexity of the process, the stages of each technology, the need for calibration, and the complexity of its post-processing shall be taken into account. As a scoring criterion, the optimal technology will be understood as the one with the fewest stages and no need for pre- or post-processing; the further away from this ideal, the worse the score.

### 2.8. Criteria Weighting Method

A selection criterion was established to decide which was the best technology. This criterion was given a score from 1 to 3 for each of the comparison factors, with 1 being the best and 3 being the worst. These comparison criteria were as follows:

- Manufacturing cost: lower cost, better score.
- Total manufacturing time: lower time per manufactured unit, better score.
- Dimensional accuracy: the more dimensionally similar the manufactured part and the original file, the better the score.

### 2.9. Moisture Content and Contact Angle

To measure the moisture content, the equipment used has been the model XY-105 MW (Ossila Ltd., Sheffield, UK), with a resolution of 0.005 g, heating at 90 °C with a halogen lamp. The contact angle has been measured in the device Ossila, with the software of Ossila V 1.2.1.3 (Ossila Ltd., Sheffield, UK) to make the photograms and the software ImageJ V1.53 T (MD, USA) to process the image and determine the contact angle. The calculation method has been based on Low Bond Axisymmetric Shape Analysis (LB-ADSA), with a constant of capillarity of 1.34 × 10^5^ m^−2^, and optimization with energy gradient. This contact angle will allow us to know the level of hydrophobicity or hydrophilicity of the material, relevant to facilitating the sterilization and avoiding infections during the surgery.

## 3. Results and Discussion

### 3.1. Results of Manufacturing Processability

**Figure 5:**
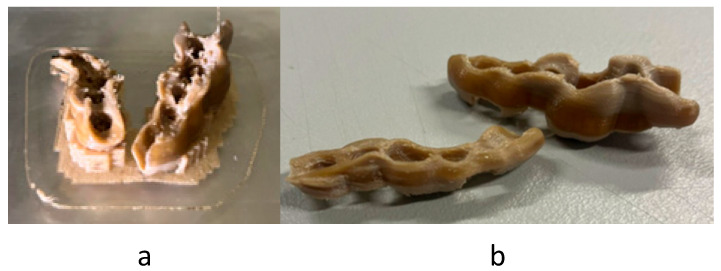
Surgical guide manufactured by MEX: just manufactured (**a**) and post-processed (**b**).

In the case of MEX, a material calibration was needed previous to the manufacturing process. In Figure 5a, the set just manufactured is shown. The parts present some melted material dots and support. The melted dots were cleaned and the support removed by q mechanical process, finally remaining as in Figure 5b. The finishing was soft enough to the touch without other machines or processes needed.

**Figure 6:**
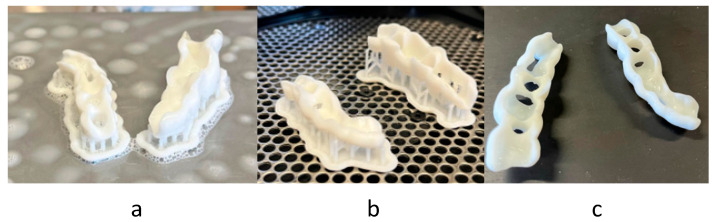
Surgical guide manufactured by VPP: just manufactured (**a**), cleaned and cured (**b**), and final set (**c**).

For VPP technology, just after manufacturing, the parts were covered with non-cured resin, so a wash with isopropyl alcohol (Figure 6a) was needed. After that, a post-curing was needed to guarantee the mechanical properties of the material (Figure 6b) and, finally, the supports were removed (Figure 6c). Compared to MEX, this technology does not require calibration, but the washing step of uncured resin adds extra time and cost.

**Figure 7:**
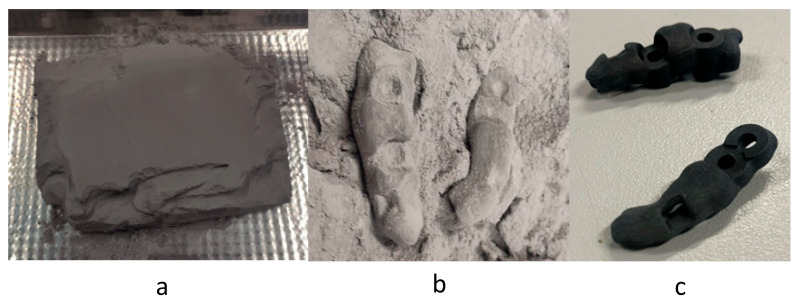
Surgical guide manufactured by PBF: block of powder (**a**), printed parts in the post-processing process (**b**), final parts (**c**).

In the case of PBF technology, when the machine just finished, the parts were retrieved from a block of agglomerated powder (Figure 7a) by mechanical means (aspiration and brushing), as shown in Figure 7b. After this, the parts were sandblasted, eliminating the small remains of excess material adhered to the parts (Figure 7c). The surface of these parts felt much rougher to the touch than with previous technologies. The configuration of the lamination parameters was almost automatic, but the preparation of the powder inside the machine before the manufacturing was too slow. Also, managing the unsintered powder after manufacturing was a complex, dangerous, and dirty process. This post-processing step required a specialized vacuum and sandblasting machines.

### 3.2. Results of Accuracy and Dimensional Study

The measurements obtained using the profile projector are compared in Table 3.

**Figure 8:**
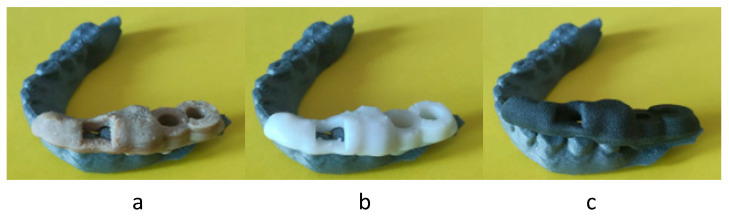
Pass/fail test results: MEX (**a**), VPP (**b**), and PBF (**c**).

From this comparison, a big discrepancy between PBF and other techniques was evidenced; thus, the exterior dimensions of the PBF part were measured, noting a considerable dimensional increase of the piece in all directions, unlike the others. Therefore, VPP technology exhibited the best dimension and accuracy, followed by MEX and PBF, which performed the worst. The additional pass/fail test confirmed these results (Figure 8): MEX and VPP passed (Figure 8a,b), while PBF failed (Figure 8c). Therefore, PBF technology could be problematic in terms of accuracy, especially in the absence of strict humidity and temperature controls when using the manufactured part. However, VPP technology achieved an almost perfect fit, while MEX technology achieved the necessary precision.

### 3.3. Manufacturing Cost of a Set of Parts

Table 4 shows the cost of use of each technology, taking into account both the main and accessory machines.

The times consumed during manufacturing are collected in Table 5.

In addition, from the slicer configuration, the volume of material used for each technology to print the same set of parts was obtained; being 6.12 cm^3^, 9.85 cm^3^, and 5.27 cm^3^ in the case of MEX, VPP, and PBF, respectively.

Taking into account the different machines used, the manufacturing costs corresponding to each technology were calculated. In this first calculation, the material cost was not considered. The cost of machine use can be seen in Table 6.

In this first comparison, it is notable that the operator time is the most significant factor, so reducing this parameter is crucial. In the case of PBF, printer preparation and the need for manually post-processing put it in the worst position, as this parameter does not depend on the printed part characteristics for most parts.

On the other hand, when comparing VPP and MEX, the latter had higher acquisition costs and electrical consumption, but less operating time was required, so both technologies are very similar in terms of cost.

### 3.4. Manufacturing Costs of Multiple Sets of Parts

**Figure 9:**
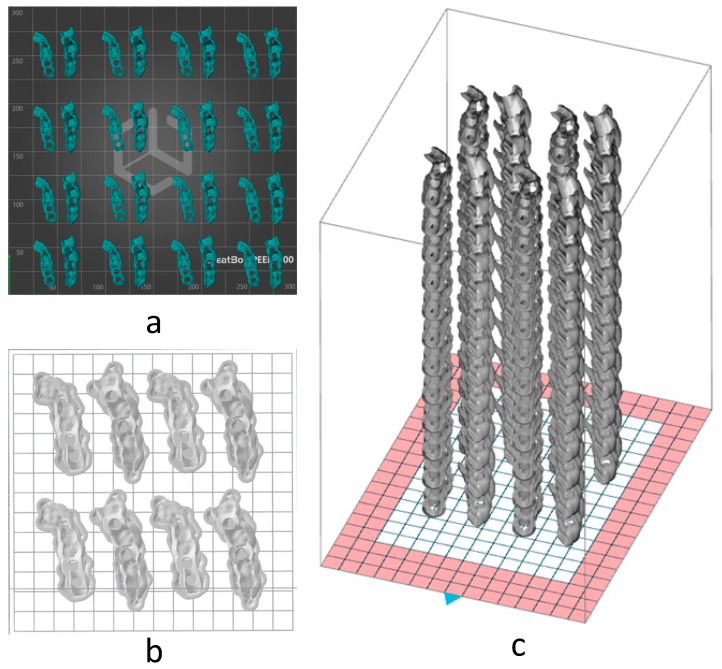
Placement of parts in the slicer software used in MEX (**a**), VPP (**b**), and PBF (**c**).

To evaluate the costs associated with the manufacturing of multiple sets of parts, using the slicer, the following configurations were obtained:

- MEX: MEX technology needs the use of supports, so only one layer of sets can be manufactured, being limited by the print area. Figure 9a shows the final arrangement. A total of 16 sets can be placed.
- VPP: This technology also needs the use of supports, so only one layer of sets was arranged, limited by the print area (Figure 9b). A total of four sets can be placed.
- PBF: This technology has the particularity of not needing supports for the manufacturing of parts, so that we can use the entire print volume of the machine. This allowed the placement of a total of 48 sets with the arrangement shown in Figure 9c.

For the calculation of the associated manufacturing costs, the needed times were evaluated (Table 7). As the operator’s time sometimes overlaps with the machine’s work, it was measured separately.

The manufacturing cost of a set is reported in Table 8.

Comparing Table 6 and Table 7, it is observable that VPP technology has a faster production time than MEX. Furthermore, despite the different number of sets produced (16 in MEX and 4 in VPP), Table 8 shows that the cost per set in VPP is less than half that of MEX. In the case of multiset manufacturing, PBF technology is in an intermediate position between the two previous technologies, but with a very high increment in the manufacturing process time.

### 3.5. Manufacturing Costs Depending on the Material

Taking into account again the manufacturing of a single set of parts and the material volumes reported in Section 3.3. The final cost of the set, including the material cost, is presented in Table 9, where different materials for each technology are compared.

Table 9 shows that the PBF technology cost, including material, is more stable compared with the other technologies. However, in PBF, the material cost is not so relevant to the final cost, so this technology remains the most expensive one.

In the case of VPP, the material cost range is small and does not greatly influence the total manufacturing cost, but it does significantly reduce the cost compared with PBF.

Finally, the price range for the MEX material is very wide, being the most inexpensive in the list in most cases (under the VPP and almost half of PBF). It is noteworthy that the material price for this technology can greatly influence the final price, as some materials can cost half as much as others, doubling the final price of the manufactured part.

### 3.6. Scores Achieved

According to the previously commented criteria, the scores obtained per technology in each analysis point are compared in Table 10. The graphical view represents the acquired scores and highlights the difference between technologies.

In this ranking, PBF consistently performs the worst in comparison, except in terms of multiset costs, where it outperforms MEX technology as long as material costs are not considered.

VPP and MEX contest the rest of the categories, with VPP clearly ahead only in terms of multiset manufacturing costs (without considering material costs). When analyzed by category, VPP is better than MEX in terms of cost references because of the technology cost, but MEX gets ahead when the material costs are included. Additionally, MEX is a simple technology; however, VPP is faster for multisets (more similar to the real situation).

### 3.7. Clinical Practice

There are several aspects to consider in relation to the use of these materials and their respective AM technologies before and during clinical practice with a surgical guide. Although there are clear differences in the mechanical properties of the three materials under study (Table 2), this is not a critical factor in this application. This is mainly due to the fact that commercial metal inserts are commonly used in the holes where drilling will take place, meaning that the polymeric material will not come into direct contact with the drill bit. These inserts are easily adaptable to any of the three materials, and the mechanical stability of the materials is enough for clinical practice. With regard to dimensional accuracy, as mentioned in previous sections, PA12 clearly performs worse than the other two materials, mainly due to moisture absorption (see Table 11). This problem is increased during the sterilization process of the surgical guide, for example, in a hot steam autoclave, where moisture absorption can affect the dimensional stability of polyamide. In any case, the option of hybrid digital manufacturing, combining additive and CNC subtractive technologies, could provide the best level of accuracy, reducing the oversize by five-axis milling. Nevertheless, this two-step process increases the cost compared with one step with AM. As the photopolymer resin is not so stable at high temperatures, the sterilization process in the autoclave should be adjusted to avoid dimensional deviations. Peek is highly resistant to hot steam sterilization processes in autoclaves, but if it is processed using MEX technology, the resulting mesostructure, which has a certain level of porosity, could hinder sterilization processes in the internal interstitial areas. For this reason, the use of MEX technology requires the use of thin layers and a good level of overlap of the deposited filament to minimize this mesostructure. Finally, the level of hydrophilicity affects the capacity for biological contamination, which can influence the efficiency of the sterilization process. In this regard, the contact angles of the three materials processed with their respective technologies have been measured, and the results are shown in Table 11. The sample with the higher contact angle and hydrophobic behavior is PA12, mainly explained because of the surface morphology of the sintered part, in contrast to the high moisture content. The other two materials resulted in having some kind of hydrophilicity, PEEK K10 (slightly below the border of 90 °) and hydrophobicity, and white resin V4 (over 90 °).

## 4. Conclusions

The same set of surgical guides (two guides manufactured together) was fabricated using MEX, VPP, and PBF manufacturing technologies. After these processes, the manufacturing costs with and without material, the times used in the entire process for one or multiple sets, and their dimensional efficiency were measured; all these criteria were rated using a scoring system that allows for a ranking of technologies by category.

Analyzing the ranking achieved by each technology, we clearly see that PBF technology is the worst in the total count and in most of the studied categories. However, MEX and VPP technologies had the best results depending on the considered aspect: both technologies have enough dimensional accuracy, and VPP has been better by a bit; however, MEX is a cleaner and easier-to-manage technology.

Although this type of tool is usually used in scheduled surgeries, manufacturing time is something to take into account. Analyzing the manufacturing time, we see that, while for urgent needs of a specific case, MEX technology is the most suitable, when surgery can be planned and the manufacturing of different patients can be grouped, VPP technology is faster.

The manufacturing cost evaluation clearly identifies VPP technology as the optimal one when we do not take into account the material; however, the correct choice of material can lower costs, making MEX technology the best.

We must also take into account that, while in VPP technology the choice of the material does not have a significant influence, in MEX technology, it is a determining factor when choosing the machine. Because some technical materials, such as PEEK, require the use of more powerful and expensive machines, this can lead to an increase in machine acquisition costs and therefore have an impact on manufacturing costs. So, with a good analysis of what material is going to be used, the correct machine selection may entail a reduction of the acquisition cost, lowering manufacturing costs for this technology.

In addition, with the appearance of new MEX equipment with faster printing capabilities, this technology could improve the manufacturing costs of multiple sets, surpassing PBF technology, and shortening the distance in the ranking with respect to VPP, being only one point different in the global count.

After this comparison, we can conclude that, although VPP technology has traditionally been the preferred choice for the manufacturing of surgical guides, MEX technology has advanced to become competitive enough, with more available materials than others. Taking into account its versatility and its greater ease of use, MEX technology appears to be the ideal one for rapid incorporation into the majority of small dental clinics. As a complement to additive manufacturing, the use of CNC subtractive manufacturing technologies allows surface defects to be corrected and dimensional accuracy to be significantly improved. This combination, known as hybrid manufacturing, is affordable for small dental clinics given the existence of low-cost additive and subtractive equipment.

## Figures and Tables

**Table 1:** Examples of applications in dentistry by AM technologies.

AM Category	Subcategory	Cases of Use
Vat Photopolymerization (VPP)	SLA	Customized implants [16] Surgical guides [21,26,27,28,29] Provisional restorations [26,30] Anatomic diagnostic models [31] Orthopedic implants [32] Occlusal splints [29]
	DLP	Diagnostic study models [33] Dental models [25,34] Surgical guides [21,27,35] Gingival masks [21] Implants [20]
Material Extrusion (MEX)		Customized implants [16] Dental models [25] Customized impression trays for edentulous jaws [31,36] Temporary crowns and bridges [28,32] Barrier membranes for bone defects [37] Flexible dental prostheses [28]
Powder Bed Fusion (PBF) Polymers and metals	SLS	Customized implants [16] Dental models [23,25] Metal caps and frameworks [26,28] Crowns and dentures [28] Titanium and titanium alloy implants [38]
	SLM	Customized implants [16] Near full density, high quality and complex geometries [16] Metal copings and frameworks [26,28] Full arch fixed dental prostheses (FAFDPs) [39] Customized titanium mesh [30] Metal primary frameworks (Ti-6-Al-4-V alloy and Co-Cr alloy) [20]
	EBM	Near full density, high quality and complex geometries [16] Full arch fixed dental prostheses (FAFDPs) [39] Open cell metal implants [18] Primary metal frameworks (Co-Cr alloy) [20] Dental implants [20]

**Table 2:** Mechanical and physical properties of the selected materials. Supplier datasheet.

	PEEK K10 (MEX)	Sinterit PA12 (PBF)	White Resin V4 (Postcured) (VPP)
Tensile E modulus (Mpa) (ISO 527)	4200–4500	1662	
Tensile E modulus (Mpa) (ASTM D 638-14)			2800
Tensile strength (Mpa) (ISO 527)	70–80	42.3	
Tensile strength (Mpa) (ASTM D 638-14)			65
Flexural Modulus (Mpa) (ISO 178)	2400–2600	1506	
Flexural Modulus (Mpa) (ASTM D 790-15)			2200

**Table 3:** Diameters and distances measured.

	Hole 1 Diameter (mm)	Hole 1 Error (%)	Hole 2 Diameter (mm)	Hole 2 Error (%)	Distance Between Holes (mm)	Distance Between Holes Error (%)
Design	5.804	0	5.805	0	8.05	0
VPP	5.437	6.3	5.578	3.9	8.063	0.2
PBF	4.185	27.9	4.332	25.4	8.566	6.4
MEX	5.197	10.5	5.219	10.1	8.109	0.7

**Table 4:** Calculation of the cost per minute of use for each machine necessary for the different technologies.

	MEX	VPP		PBF		
	Printer	Printer	Curing	Printer	Sandblaster	Vacuum Cleaner
Electrical consumption of the machine (kW/h)	4.6	0.065	0.15	1	-	1.1
Electrical cost per minute (EUR)—Cem	0.01917	0.00027	0.00063	0.00417	0	0.00458
Machine price (EUR)	17,500	7750	1150	18,500	3530	4250
Maintenance and others (EUR)	1750	775	115	1850	353	425
Depreciation of machine and maintenance per minute (EUR)—CU	0.01082	0.00436	0.00065	0.01040	0.00198	0.00239

**Table 5:** Time consumed for manufacturing.

	MEX		VPP			PBF			
	Operator	Production	Operator	Production	Curing	Operator	Production	Dust Suction	Sandblaster
Previous preparation (min)	10		10			10			
Extraction (min)	1		10			30			
Manufacturing (min)		80		144			390		
Post-processing (min)	15		10		30	15		10	5
Total by process (min)	26	80	30	144	30	55	390	10	5
Total by technology (min)	106		204			445			

**Table 6:** Manufacturing costs by technology (excluding material cost).

	MEX	VPP		PBF		
	Printer	Printer	Curing Machine	Printer	Sandblaster	Vacuum Cleaner
Machine use cost (EUR/min)	0.011	0.004	0.0007	0.010	0.002	0.002
Electrical cost per minute (EUR/min)	0.019	0.0003	0.0006	0.004	0	0.005
Manufacturing time (min)	80	144	30	390	15	15
Machine use and electrical costs (EUR)	2.399	0.705		5.816		
Operator cost per minute (EUR/min)	0.2	0.2		0.2		
Operator time (min)	26	30		55		
Operator cost (EUR)	5.2	6		11		
Total manufacturing cost (EUR)	7.60	6.70		16.76		

**Table 7:** Manufacturing times for multiple sets (in minutes).

	MEX		VPP			PBF			
	Operator	Production	Operator	Production	Curing	Operator	Production	Dust Suction	Sandblasting
Previous preparation time (min)	10		10			10			
Extraction time (min)	1		10			30			
Manufacturing time (min)		2572		210			5970		
Post-processing time (min)	184		33		98	544		363	181
Total by process (min)	195	2572	53	210	98	584	5970	363	181
Total by technology (min)	2767		361			6554			

**Table 8:** Manufacturing cost per set in a multiple-set manufacturing (excluding material cost).

	MEX	VPP		PBF		
	Printer	Printer	Cure	Printer	Sandblaster	Vacuum Cleaner
Machine use cost (EUR/min)	0.01082	0.00436	0.00065	0.01040	0.00198	0.00239
Electrical cost per minute (EUR/min)	0.01917	0.00027	0.00063	0.00417	0	0.00458
Manufacturing time (min)	2572	210	98	5970	363	181
Machine use and electrical costs (EUR)	77.137	1.097		88.961		
Operator cost per minute (EUR/min)	0.2	0.2		0.2		
Operator time (min)	195	53		584		
Operator cost(EUR)	39	10.6		116.8		
Total manufacturing cost (EUR)	116.14	11.70		205.76		
Manufacturing cost per set (EUR/set)	7.26	2.92		4.29		

**Table 9:** Manufacturing cost of a set of parts depends on the material used.

Technology	Material	Material Cost per Volume (EUR/cm^3^)	Material Cost per Set (EUR/Set)	Manufacturing and Material Costs (EUR/Set)	Average Cost (EUR/Set)
MEX	BASF PLA PRO1 Ultrafuse	0.0592 [50]	0.3621	7.96	9.24 EUR/set
MEX	PP PPprint 721	0.0573 [51]	0.3507	7.95	
MEX	MedPhen PEEK Ketaspire	0.9126 [52]	5.5851	13.18	
MEX	FLXR Filament PEN–Polietileno naftalato	0.2638 [53]	1.6145	9.21	
MEX	ABS Medical Smartfil	0.0497 [54]	0.3042	7.90	
VPP	Resina KeyDenture Try-In	0.3594 [55]	3.5403	10.24	10.08 EUR/set
VPP	Resina MED413–Loctite 3D	0.5160 [56]	5.0826	11.79	
VPP	Dental IBT–HARZ Labs	0.1536 [57]	1.5133	8.22	
PBF	Sinterit PA12 Industrial	0.0814 [58]	0.4288	17.19	17.34 EUR/set
PBF	Sinterit PA12 Smooth	0.1104 [59]	0.5818	17.34	
PBF	Sinterit PBT Optimal	0.1143 [60]	0.6022	17.36	
PBF	Sinterit PP	0.1350 [61]	0.7114	17.47	

**Table 10:** Scores per technology.

Parameter	MEX	VPP	PBF
Single manufacturing cost	2	1	3
Multiset manufacturing cost	3	1	2
Manufacturing a single set cost including material	1	2	3
Single manufacturing time	1	2	3
Multiple manufacturing times	2	1	3
Manufacturing processability	1	2	3
Dimensional accuracy	2	1	3
Total scores	12	10	20
Graphical view			
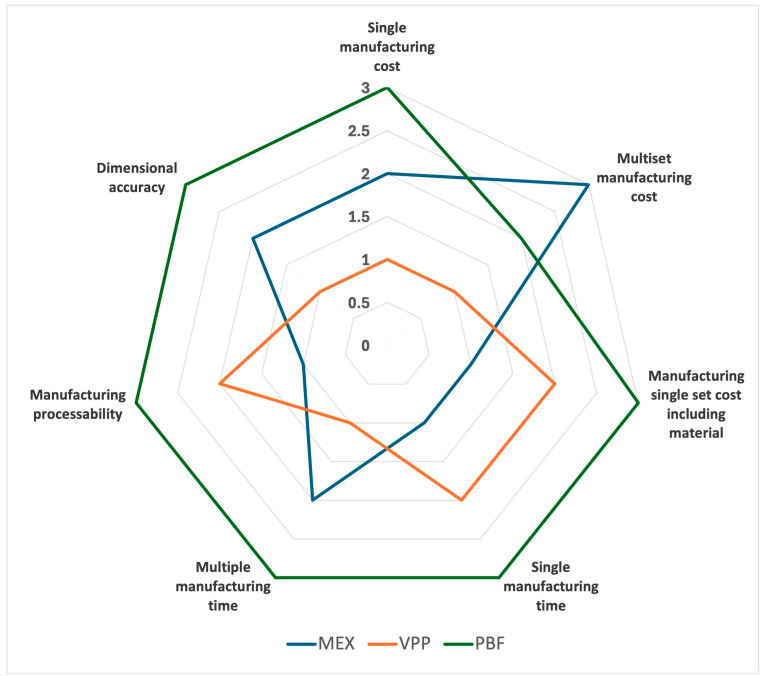			

**Table 11:** Contact angle and moisture content of the samples.

	Contact Angle (°)	Moisture Content (%)
PEEK K10 (MEX)	85.60	0.16
Sinterit PA12 (PBF)	138.23	1.23
White Resin V4 (VPP)	99.54	0.22

## Data Availability

The original contributions presented in this study are included in the article. Further inquiries can be directed to the corresponding author.
